# Genome‐Assisted Breeding of an Elite Sterile Line From Restorer Line for Hybrid Rice

**DOI:** 10.1111/pbi.70367

**Published:** 2025-09-13

**Authors:** Yu Fang, Qianlong Li, Qi Feng, Ahong Wang, Hui Wang, Yunhu Zhang, Guoli Zheng, Yunhai Kang, Dilin Liu, Huijiang Xie, Guixiang Zhou, Jing Yang, Conghe Zhang, Longjiang Fan

**Affiliations:** ^1^ Institute of Crop Science and Institute of Bioinformatics Zhejiang University Hangzhou China; ^2^ Shanghai ZKW Molecular Breeding Technology Co. Ltd Shanghai China; ^3^ Win‐All Hi‐Tech Seed Co. Ltd/Key Laboratory for New Variety Creative of Hybrid Rice Ministry of Agriculture and Rural Affairs Hefei China; ^4^ National Center for Gene Research, CAS Shanghai China; ^5^ Rice Research Institute Guangdong Academy of Agricultural Sciences Guangzhou China

**Keywords:** breeding, genome, hybrid rice, sterile line

Hybrid rice breeding is an important technology, which has achieved remarkable results over the past decades by breeders' efforts in breaking down inter‐subspecies barriers and realising the aggregation of superior genes (Huang et al. 2016; Zheng et al. 2023). However, in the context of climate change and population growth, there is an urgent need to harness cutting‐edge technologies such as genomics to enable the precise and efficient breeding of new crop varieties (Khaipho‐Burch et al. 2023; Tian et al. 2021). An effective approach is to draw on past breeding experiences to develop new theoretical methods for guiding the sustainable development of crop breeding (Huang et al. 2022). By leveraging advances in genomics, breeders can analyse complementary advantageous genes in parental lines of hybrid rice to develop novel sterile lines in three‐line hybrid rice breeding. In three‐line hybrid rice systems, the inherent constraints of the restorer–maintainer relationship present substantial challenges for the development of sterile lines from restorer lines. Quan9311A is an elite three‐line sterile line developed from a two‐line restorer line through traditional breeding and molecular marker‐assisted selection (MAS) based on quantitative trait loci (QTLs) information, exhibiting outstanding overall agronomic traits. It is currently being promoted in agricultural productions with a large area, but further improvements are needed in blast resistance, grain quality and plant architecture. In this study, we reported the genome‐assisted breeding of an elite sterile line (Huke1A) using the restorer line (YR0822) as a parental line (a starting point) for hybrid rice to enhance Quan9311A (Figure 1a).

**FIGURE 1:**
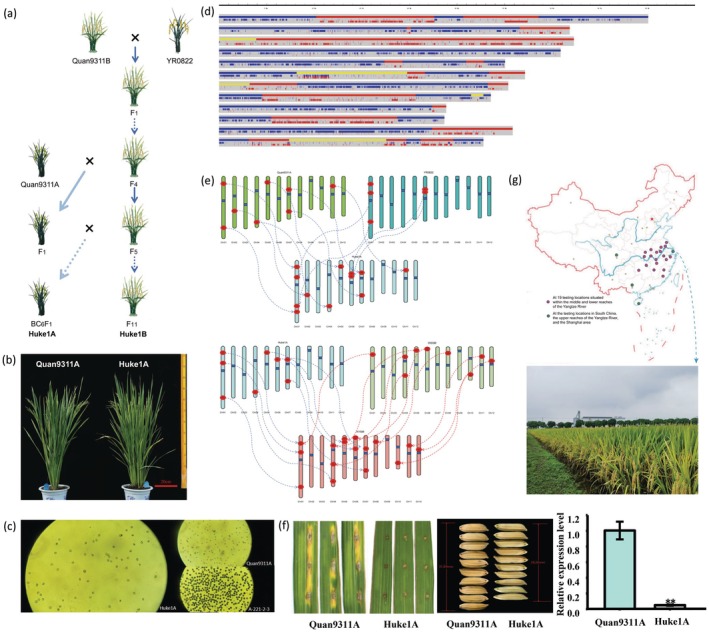
Genome‐assisted breeding of an elite sterile line Huke1A. (a) Breeding roadmap of the elite sterile line Huke1A through pyramiding superior genes. (b) Comparison of plant phenotypes between Huke1A and parental Quan9311A. (c) Pollen fertility analysis of Quan9311A, Huke1A and A‐221‐2‐3. A‐221‐2‐3 is a line from which the restorer gene has not been removed. (d) Genomic profiling of Huke1A. The Huke1A genomic segments delivered from two parental lines (blue: Quan9311A; red: YR0822; yellow: heterozygous). (e) Superior alleles inherited by Huke1A and its hybrid KYSM. Top part, the alleles marked with arrows within circles are inherited by Huke1A from Quan9311A and YR0822. Bottom part, the alleles marked with arrows within circles are aggregated by KYSM from Huke1A and its restorer WSSM. (f) Comparison of rice blast resistance, grain size and *GS5* expression level between Quan9311A and Huke1A. Left, rice blast resistance of Quan9311A and Huke1A, 7 days post‐inoculation with punch inoculation. Middle, grains of Quan9311A and Huke1A. Right, the relative expression level of *GS5* in the seeds of Quan9311A and Huke1A at 22 days post‐pollination (g) The distribution of field test sites for KYSM in 2 years (2021–2022) with its plant phenotype observed in Shanghai in 2023.

We systematically collected the genome sequences of over 500 parental lines utilised in hybrid rice breeding (Gu et al. 2023). Using these sequences, we analysed the genomic background of the parental lines, focusing on the causative variants (also called as QTNs, short for quantitative trait nucleotides) in Quan9311A using RiceNavi (Wei et al. 2021). For example, compared with 9311, the restorer alleles of *Rf3* and *Rf6* were eliminated, and the sterile alleles of *WA352c*, which belong to wild abortive cytoplasmic male sterility, were introduced (Qianlong et al. 2023). Based on the phenotypic and genomic information of Quan9311A and our aims of enhancing its resistance to rice blast, lodging resistance and grain quality, we selected the restorer line YR0822 from the collection of parental lines. YR0822 harbours the target traits and a complementary genomic background to Quan9311A, making it an ideal donor parent for trait introgression.

Quan9311B (the maintainer line of Quan9311A) was firstly hybridised with YR0822. In the F_3_ generation, a larger segregating population was created (Figure 1a). In over 2100 individuals of the population, 67 lines with field traits most similar to Quan9311B were selected for test‐crossing and backcrossing. During this process, the information of QTNs for the blast resistance gene *Pi2* and grain quality gene *GS5* was systematically integrated to minimise reliance on labour‐intensive phenotypic evaluations such as field‐based blast resistance assays and grain quality testing. After acquiring high‐coverage genomic data for both parents, we performed low‐coverage sequencing on a subset of the offspring for background selection, rather than conducting DUS (Distinctness, Uniformity and Stability) tests. Moreover, to enable efficient screening of multiple key genes, targeted capture via multiplex PCR was also implemented to develop a preliminary panel comprising 17 QTNs underlying several agronomic traits. A single‐tube operation was used to capture multiple sequence fragments of Quan9311A‐related gene loci. These fragments were then directly incorporated into a sequencing library, compatible with existing sequencing platforms, enabling a fast, convenient and cost‐effective means of obtaining information on multiple genes. Through the integration of genomic and field‐based selections, iterative optimisation yielded the three‐line sterile line Huke1A, which retains the overall agronomic performance of Quan9311A while exhibiting enhanced blast resistance, superior grain quality and optimised plant architecture (Figure 1a,b). The sterile line demonstrated superior comprehensive traits, stable agronomic performance and consistent male sterility (Figure 1c), making it suitable for commercialisation. Genomic analysis using SEG‐map (Huang et al. 2009) revealed 59.3% ancestry from parental line Quan9311A, 32.8% from YR0822 and 7.9% ‘heterozygous’ regions (Figure 1d). Except for a region on chromosome 6, all the ‘heterozygous’ regions belong to the identity‐by‐state segment between Quan9311A and YR0822. Successful introgression of *Pi2* (blast resistance) and *GS5* (grain quality) was achieved, while the restorer gene *Rf3* from YR0822 was eliminated, and cytoplasmic male sterility, conferred by the *WA352c* allele, was inherited from Quan9311A (Figure 1e). To evaluate the breeding value of Huke1A, it was crossed with an elite restorer line WSSM (Wushansimiao). Two‐year multi‐location trials across 19 geographically diverse locations in the middle‐lower Yangtze River basin demonstrated that the Huke1A/WSSM hybrid (KYSM), compared with the Quan9311A/WSSM hybrid (QYSM), exhibited a 14.5% reduction in the blast resistance index (indicating enhanced disease resistance), a one‐level improvement in rice quality (reaching the national standard's optimal grade) (Figure 1f) and a 2.4 cm decrease in plant height (Figure 1g).

Beyond the Huke1A/WSSM hybrid, a series of new hybrid rice varieties were developed using Huke1A as the parental line, demonstrating the translation of genomic insights into practical breeding applications. Our results indicated that genomics not only enhanced breeding efficiency and reduced costs—though this cost–benefit may vary in regions with minimal labour expenses—but more critically, enabled data‐driven breeding strategies. These findings are expected to encourage broader adoption of genomics in addressing complex breeding challenges. We plan to further refine Huke1A by leveraging recent insights into epistatic interaction networks and gene regulatory modules of rice (Wei et al. 2024) and by integrating artificial intelligence, genome editing and optical technologies to advance next‐generation rice varietal development.

## Author Contributions

Longjiang Fan, Conghe Zhang and Yu Fang designed the research; Yu Fang, Qianlong Li, Ahong Wang, Hui Wang, Yunhu Zhang, Guoli Zheng, Yunhai Kang, Dilin Liu and Guixiang Zhou performed the experiments; Yu Fang, Qi Feng, Huijiang Xie, Jing Yang and Longjiang Fan analysed the results and wrote the manuscript; all authors thank Prof. Bin Han and Ms. Qin Zhang for their guidance and support of this study.

## Conflicts of Interest

The variety Huke1B has been awarded the Certification of Plant Variety Rights with the registration number CNA20201002638 and is owned by Shanghai ZKW Molecular Breeding Technology Co. Ltd. KYSM has been awarded the Certification of National Market Access with the registration number GSD20233095 and is owned by Shanghai ZKW Molecular Breeding Technology Co. Ltd and Win‐all Hi‐tech Seed Co. Ltd. The applications for the Plant Variety Rights Certificate of Huke1A and KYSM remain under review.

## Data Availability

The raw DNA sequencing data of the Huke1A genomes used in this study are deposited in the National Center for Biotechnology Information (NCBI) BioProject database repository, accession number PRJNA1307643.
